# Stakeholder-Perceived Needs in Early Community Nursing Implementation: A Qualitative Study

**DOI:** 10.3390/nursrep16070228

**Published:** 2026-06-30

**Authors:** Cornelia Feichtinger, Helmut Beichler, Minna Tiainen, Igor Grabovac

**Affiliations:** 1 Center for Applied Nursing Research, Department of Applied Nursing Science, University of Applied Sciences Campus Vienna, 1100 Vienna, Austria; 2 Vienna Healthcare Group, School of Nursing, University of Applied Sciences Campus Vienna, 1100 Vienna, Austria; helmut.beichler@hcw.ac.at; 3 Department of Nursing, Faculty of Health and Social Care, Tampere University of Applied Sciences (TAMK), 33520 Tampere, Finland; minna.tiainen@tuni.fi; 4 Department of Social and Preventive Medicine, Centre for Public Health, Medical University of Vienna, 1090 Vienna, Austria

**Keywords:** community nursing, preventive care, stakeholder perspective, social needs

## Abstract

**Background/Objectives:** Health systems across Europe are increasingly challenged by population ageing, multimorbidity, and persistent inequities in access to care. Community Nursing has emerged as a promising approach to strengthening preventive, community-based services, yet evidence on stakeholder-perceived needs during early implementation remains limited. This study aimed to explore how different types of needs are perceived and articulated during the early implementation of Community Nursing in Austria. **Methods:** An interpretive descriptive qualitative study was conducted as part of an Austrian Community Nursing pilot project. Semi-structured interviews were carried out with eleven stakeholders, including informal caregivers, network partners (e.g., local healthcare providers), and local political decision-makers. Data were analyzed using qualitative content analysis, guided deductively by Bradshaw’s Taxonomy of Needs and complemented by inductive sub-category development. Interviews were conducted via telephone or video call (Zoom) and ranged from approximately 30 min to an hour. **Results:** Normative needs reflected expectations for preventive services, continuity of care, advocacy, and sustainable organizational structures. Felt needs centered on trust, emotional security, and relational continuity. Expressed needs became visible through active use of Community Nursing services, including preventive programs, transitional care support, and administrative navigation. Comparative needs highlighted geographic inequities and differences between municipalities with and without access to Community Nursing. Across stakeholder groups, concerns regarding long-term financing and sustainability were prominent. **Conclusions:** The findings suggest that Community Nursing addresses multiple stakeholder-perceived needs simultaneously, particularly by providing relational, accessible, and preventive support. However, sustained impact depends on stable funding and systemic integration beyond pilot phases. These results offer transferable insights for the development and scaling of community-based nursing models in ageing societies.

## 1. Introduction

Health systems across Europe and other high-income countries are increasingly challenged by population ageing, rising multimorbidity, and growing demands for long-term and community-based care [1,2]. These developments are accompanied by structural pressures on healthcare systems, including rising healthcare expenditure and persistent fragmentation between sectors of care [3,4]. In many settings, historically hospital-centred models of service delivery struggle to respond adequately to the everyday health-related needs of community-dwelling older adults, particularly those with complex or chronic conditions [5,6]. Strengthening primary and community-based care has therefore become a central policy objective, with community nursing increasingly positioned as a key strategy to promote healthy ageing, prevention, and continuity of care close to people’s living environments [7,8].

Research on community and public health nursing highlights a broad range of intended functions, including health promotion and prevention, care coordination and navigation, relational continuity, and the reduction in health inequities [9,10,11]. Empirical studies indicate that continuity of care and sustained professional relationships are associated with improved health outcomes, reduced hospitalisations, and lower healthcare costs, particularly for older populations and people with chronic conditions [12,13,14,15]. In addition, navigation and case management approaches have been shown to support access to services and coordination across health and social care systems [16,17]. Relational and person-centred aspects of care, including trust, communication, and relationship-centred practices, are increasingly recognised as central components of high-quality nursing care for older adults [18,19,20].

Despite this growing body of evidence, much of the existing literature focuses on established models of care or on outcomes of mature services. Comparatively less attention has been paid to early phases of implementation, during which new community nursing roles, responsibilities, and service structures are still being defined. Early implementation phases are critical, as they shape how services are perceived, utilised, and further developed over time. During these phases, different stakeholder groups may articulate distinct and sometimes competing needs and expectations. If such needs are not adequately recognised or aligned, barriers to access and utilisation may persist or be reinforced, particularly for older adults facing mobility limitations, rural residence, or other structural disadvantages [21].

In Austria, community nursing was introduced in 2022 as an European-funded pilot initiative within a traditionally fragmented and hospital-centred healthcare system [22,23]. The pilot regions represent an early-stage implementation setting in which community nursing roles, target groups, and organisational arrangements are still evolving. Pilot sites included both small rural municipalities and larger towns, providing some variation in settlement context across the study. Pilot sites included both small rural municipalities and larger towns, providing some variation in settlement context across the study. Rather than serving as a nationally representative model, this context provides a relevant case example for examining how stakeholder-perceived needs become visible during the introduction of community nursing in an established but transforming care system. Insights derived from this setting may therefore inform community nursing implementation efforts in comparable health system contexts.

Within health services research, needs are not understood solely as objective deficits or measurable service gaps, but as socially constructed, negotiated, and context-dependent phenomena shaped by professional norms, lived experiences, institutional structures, and power relations. This understanding extends beyond Bradshaw’s original taxonomy, which provides a useful analytical structure but does not fully capture the relational, fluid and situated character of needs as they are lived and articulated by different actors over time. Different stakeholder groups may therefore define and prioritize needs differently depending on their social position, responsibilities, and experiences within the healthcare system [24].

While prior research has examined the functions and outcomes of established community and public health nursing models, comparatively few studies have addressed how multiple stakeholder groups perceive and negotiate needs simultaneously during the early implementation of a new community nursing service. This gap is particularly relevant given that early implementation phases shape how services are subsequently used, adapted, and sustained. The aim of this study was to explore stakeholder-perceived needs in the early implementation of community nursing using a qualitative approach. By analysing perspectives from multiple stakeholder groups, including (1) informal caregivers, (2) network partners such as local healthcare providers, and (3) local political decision-makers, through a theoretically informed needs framework, the study seeks to contribute to a deeper understanding of how different types of needs are articulated and negotiated during early community nursing implementation.

## 2. Materials and Methods

### 2.1. Study Design

This study used a qualitative design and applied qualitative content analysis to explore stakeholder-perceived needs in the early implementation of Community Nursing. The analysis was theoretically informed by Bradshaw’s Taxonomy of Social Need, distinguishing normative, felt, expressed, and comparative needs [24]. The manuscript was prepared in accordance with the COREQ (Consolidated Criteria for Reporting Qualitative Research) guidelines; the completed checklist is provided in the Supplementary Materials. Methodologically, the study followed an interpretive descriptive design, which combines a theory-informed analytical lens with a practice-oriented, descriptive approach aimed at generating knowledge applicable to nursing practice and policy. This orientation is consistent with the combined deductive–inductive analytical strategy applied in this study.

### 2.2. Setting and Context

The study was conducted within the context of the Austrian Community Nursing pilot programme, introduced in 2022. Data collection was conducted during the early implementation phase, when community nursing roles, local networks, and service pathways were still in the process of development.

To analyse needs in a systematic and differentiated manner, this study draws on Bradshaw’s Taxonomy of Social Need, which distinguishes between normative needs, defined by professional standards; felt needs, experienced and articulated by individuals; expressed needs, translated into demands or service use; and comparative needs, identified through comparisons between populations or regions [24].

In the present study, Bradshaw’s taxonomy is not understood as an empirical outcome, but as a theoretical framework that structures the analysis. It provides an analytical lens through which stakeholder perspectives on community nursing can be examined and compared, allowing different dimensions of need to be made visible during early implementation. The taxonomy thus supports a theory-informed interpretation of qualitative data rather than a categorisation derived inductively from the material. This framework has been applied in health services research to explore discrepancies between professional assessments, lived experiences, and service provision, and to support structured needs assessments at population and service levels [25]. As such, it offers a suitable analytical lens for examining stakeholder perspectives in early phases of community nursing implementation.

### 2.3. Participants and Recruitment

A purposive sampling approach was used to recruit participants able to provide insights into the implementation and experience of Community Nursing from three stakeholder perspectives, namely (1) informal caregivers, (2) network partners, such as local healthcare providers, and (3) local political decision-makers involved in or informed about local implementation processes.

Recruitment was conducted via existing project networks and targeted contacts in pilot settings. Inclusion criteria were (a) belonging to one of the stakeholder groups and (b) direct experience with Community Nursing implementation and/or collaboration during the pilot phase. All participants were informed about the study aims, confidentiality, and voluntary participation. Specifically, contacts for informal caregivers, network partners, and local political decision-makers were facilitated by community nurses working in the pilot project, with whom the first author had previously conducted separate interviews within the same project. While these community nurses had no influence on interview content, their role in facilitating access to participants is acknowledged as a potential source of selection bias, as discussed further in the Strengths and Limitations section.

In total, 11 individuals participated: five informal caregivers, three network partners, and three local political decision-makers. Participants were aged 32–76 years (mean 54.5, SD ± 12.56) and included nine women and two men. Interviews were conducted between October 2023 and March 2024. This study was designed from the outset as an exploratory investigation of an early-stage pilot project; it was not designed to achieve theoretical saturation independently within each of the three stakeholder subgroups. Sampling aimed to include information-rich cases across stakeholder groups, and data collection and coding proceeded iteratively, with each interview coded before the next was conducted. From the ninth interview onward, no substantially new subcategories emerged; two further interviews were subsequently conducted to confirm this pattern, supporting the adequacy of the sample for the exploratory aim of the study. Table 1 presents anonymized characteristics of all 11 participants, including gender, age, stakeholder group, interview mode, and interview duration.

### 2.4. Data Collection

Data were collected using semi-structured interviews conducted via telephone or online video calls (Zoom), depending on participant preference. Three distinct interview guides were developed and tailored to the perspectives of (1) informal caregivers, (2) network partners, and (3) local political decision-makers. Guides included open questions on experiences with Community Nursing, perceived needs related to health and care in the community, expectations toward Community Nursing, and perceived gaps in services and structures. All interview guides were developed collaboratively among several members of the research team prior to data collection. All 11 interviews were conducted by the first author, a nursing researcher, who had no prior personal relationship with any of the participants. Interviews ranged from 28 min 57 s to 1 h 7 min 27 s in duration (see Table 1). After each interview, the first author recorded reflective notes in a research diary, documenting contextual observations, methodological decisions, and any notable circumstances.

Most participants opted for telephone interviews, mainly due to familiarity and accessibility. Interviews were digitally audio-recorded with consent and transcribed verbatim. Identifying information was removed during transcription, and transcripts were pseudonymised for analysis. Specifically, place names (e.g., municipalities) and names of third parties mentioned by participants (e.g., physicians or other professionals) were removed or replaced with generic placeholders prior to coding. To further reduce the risk of re-identification, given the small sample size, local political decision-makers are described in this manuscript without reference to specific functions (e.g., mayor) or municipality, given the potential identifiability of individuals holding such roles in small communities. We did not observe meaningful differences in the depth, richness, or openness of responses between telephone and Zoom interviews. If anything, the telephone format appeared to be experienced as particularly comfortable and familiar by several participants, notably among older informal caregivers, for whom this mode may have lowered communicative barriers rather than limited the depth of disclosure.

### 2.5. Data Analysis

Transcripts were analysed using MAXQDA 22.7.0. We applied a content-structuring qualitative content analysis approach [26]. The analytic strategy combined deductive and inductive steps: (1) Deductive framework: Bradshaw’s taxonomy was used to define four a priori main categories (normative, felt, expressed, comparative needs) [24]. (2) Secondly, all transcripts were coded to assign meaning units to one of the four main categories. (3) Afterwards, inductive subcategories emerged, and within each main category, inductive subcategories were developed from the data through iterative coding rounds [26]. (4) Finally, iterative refinement took place by comparing cases and revisiting category definitions. Analytical memos were used to document coding decisions and emerging interpretations. To avoid overclaiming, we use the term information-rich sampling and describe that during the final interviews, no substantially new subcategories emerged, supporting the adequacy of the sample for the study aim. Coding was conducted by the first author. To enhance interpretive rigor, the alignment of inductively developed subcategories with Bradshaw’s deductive main categories was subsequently discussed and reflected upon together with a co-author (H.B.) as a form of peer debriefing. Analytical memos documenting coding decisions were maintained throughout in MAXQDA, providing an audit trail of the analytical process. Member checking (participant validation of the findings) was not conducted.

### 2.6. Ethical Considerations

The study received ethical approval from the ethics committee of the University of Applied Sciences FH Campus Wien (EK 113/2023, 21 April 2023). All participants provided informed consent prior to the interviews. Participation was voluntary and could be withdrawn at any time. Confidentiality and anonymity were ensured through pseudonymization and careful handling of potentially identifying contextual information.

## 3. Results

**Figure 1:**
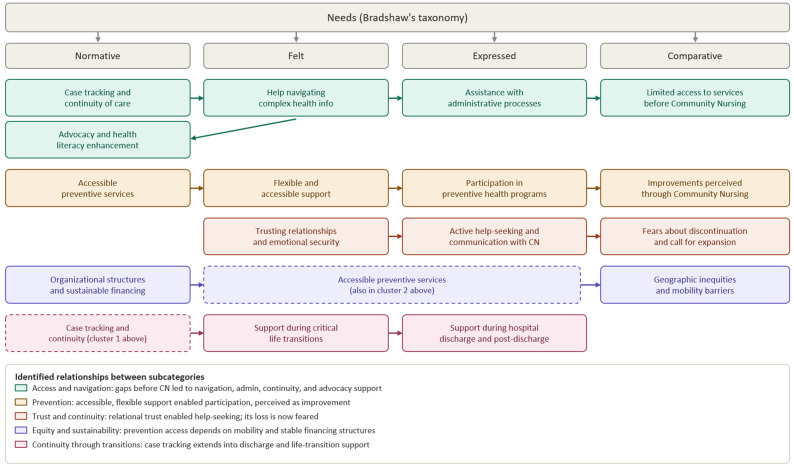
Analytical structure of stakeholder-perceived needs in Community Nursing based on Bradshaw’s taxonomy. Dashed borders indicate subcategories that overlap with a cluster presented elsewhere in the figure (cross-referenced rather than duplicated).

The analysis was structured using Bradshaw’s Taxonomy of Needs as a predefined analytical framework [24]. The four need dimensions (normative, felt, expressed, and comparative needs) served as deductive main categories, within which inductive subcategories were developed based on the empirical material. An overview of the analytical framework and category structure is presented in Figure 1, highlighting three identified relationships among subcategories across need dimensions, in addition to the full category structure.

**Normative Needs**

Normative needs reflected expert- and system-level expectations regarding services and structures considered necessary to maintain the health, independence, and well-being of older adults. These needs were articulated primarily by network partners and local political decision-makers and concerned structural, organizational, and professional expectations guiding the implementation of Community Nursing.

**Need for Accessible Preventive Services**

Stakeholders framed preventive services not merely as additional support, but as a corrective response to perceived deficiencies in existing health and social care structures. Expectations toward Community Nursing reflected a broader shift from reactive and episodic care toward anticipatory, relationship-based support embedded within local communities.

One political decision-maker described the limited availability of services within the community:

*“[…] it wasn’t really a classic community issue before, we have social counselling once a month, a lady comes to us, she has office hours, I think from two to three hours, as I said, that’s kind of the intention from the desk, you can get brief advice there, but there is no home visit […]”*

(A8, 142–147)

Participants described the absence of consistent and proactive preventive services as a critical structural gap that Community Nursing was expected to address systematically. Community Nursing was seen as a mechanism to offer not only reactive but also proactive support through ongoing, structured contact with clients. These accounts suggest that prevention was understood less as a narrowly clinical intervention and more as a continuous social and relational process aimed at maintaining independence and avoiding crisis-driven care trajectories.

In line with the preventive mandate, stakeholders emphasized the importance of early interventions aimed at maintaining older adults’ independence:

*“Yes, this is prevention for older people, so that they can stay in their own homes for longer and are well looked after.”*

(A11, 20–22)

This perspective reflects an understanding that community health services must be embedded into daily life, rather than activated only during acute crises.

**Need for Case Tracking and Continuity of Care**

A second normative expectation concerned ongoing case tracking and continuity of care. Stakeholders emphasized that community nurses should maintain active, long-term relationships with clients to enable early identification of health deterioration and timely intervention. Informal caregivers described how community nurses followed up proactively:

*“Well, and then we made sure that someone was always there, I also called and checked whether she was doing well anyway, if I couldn’t drive up, I called, dad called, the community nurses checked regularly, the legal guardian also checked, so we took turns, I’d say, on a regular basis.”*

(A10, 549–554)

The regular, proactive engagement created a safety net for vulnerable older adults and their families. This emphasis on continuous rather than episodic contact was considered essential for building effective preventive care structures.

Another caregiver highlighted how continuity facilitated better understanding of client needs:

*“But what really impressed me was that they really visited my mum regularly, that they understood her needs, and what also helped me, for example, was that they kept talking to me about what the situation was like now, because my mum is, I think, from my point of view, in the onset of senile dementia.”*

(A2, 81–86)

Thus, normative needs included not only the availability of services but also the assurance of sustained relationships over time. This account is revisited below under Felt Needs, where the same regular contact is examined for its emotional rather than structural significance.

**Need for Advocacy and Health Literacy Enhancement**

Another key normative need related to the role of community nurses as advocates for their clients. Stakeholders highlighted that older adults and their caregivers often face overwhelming challenges when navigating the healthcare system, particularly regarding information overload.

From the client’s perspective, the need for someone to mediate complex medical information was evident:

*“[…] the doctor explained so much to me, I always listened and then I was back at home ‘what did he say [spoken with question intonation]’, there are situations where you simply can’t process this wealth of information, and I think when you get even older it becomes even more difficult.”*

(A2, 398–402)

Stakeholders thus viewed it as a core responsibility of community nurses to empower clients through clear communication, education, and navigation support and addressing a normative need for greater health literacy.

Additionally, advocacy extended to system-level navigation, such as supporting applications for care allowance:

*“[…] that someone got a rejection when the care allowance was increased and no longer knew their way around, so in the past we just made sure that we got on somehow, and now it’s ideal, because now I can say the community nurses are just the right people.”*

(A7, 263–266)

Community nurses were seen as crucial intermediaries who could advocate effectively on behalf of clients within a fragmented health and social care system.

**Need for Organizational Structures and Sustainable Financing**

Finally, stakeholders identified organizational and financial stability as indispensable for the success and expansion of Community Nursing.

Many participants noted that municipalities alone could not sustain the service structure:

*“So I believe that Community Nursing should not necessarily remain at the municipal level, it should be raised to regional level (…) it could be that the social welfare association specifies something, that is a construct where we say that would basically fit.”*

(A8, 311–322)

A political decision maker stated that:

*“If so, then I certainly see this with the social welfare associations, because we as municipalities are actually not the right, what does not mean the right contact person, is perhaps the wrong expression now, but it should definitely continue and already be organized via the social welfare associations.”*

(A11, 373–376)

Concerns about financing were expressed across multiple interviews. Participants feared that without secured funding, the gains achieved through the pilot project might be lost:

*“[…] when the funding project is over, I can’t go here as a municipality and say ‘I’ll take 100,000 euros a year to continue operating this service […]’“*

(A8, 374–376)

A political decision maker stated that:

*“Then somewhere along the line someone said ‘yes, the municipalities have to help finance it’, yes, but if we really go down that route, then you have to be honest and say that we’ll have a problem or we’ll get a problem.”*

(A11, 401–404)

Participants further framed Community Nursing as a broader systemic investment, arguing that preventive and community-based support could reduce institutional care utilization and associated long-term costs.:

*“So, what you can say quite clearly, I believe that with this service,* i.e., *what Community Nursing costs on the one hand, you certainly save several times over on the other in terms of nursing services.”*

(A8, 366–369)

In this sense, the normative need for sustainable financial support was framed not only as a budgetary issue but as a broader investment in the efficiency and equity of Austria’s healthcare system.

**Felt Needs**

Felt needs were strongly shaped by experiences of relational continuity and emotional reassurance. Participants did not describe Community Nursing primarily in terms of technical nursing tasks but as a dependable relational presence that reduced uncertainty and strengthened perceived security in everyday life.

**Need for Trusting Relationships and Emotional Security**

The establishment of trusting, empathetic relationships was repeatedly emphasized as one of the most significant sources of satisfaction and well-being among participants. Community nurses’ repeated visits, personal engagement, and familiarity with individual life situations fostered a sense of safety, emotional security, and relational trust.

One participant expressed how regular interactions built a strong foundation of understanding and emotional closeness, beyond the continuity-of-care function discussed above under Normative Needs:

*“[…] but what really impressed me was that they really visited mum regularly, that they understood her needs, and what also helped me, for example, was that they kept talking to me about what the situation was like now […]”*

(A2, 81–85)

Another participant emphasized the value of the professional knowledge community nurses brought, and how quickly trust developed:

*“[…] because they simply have so much background knowledge, some of them have worked in hospitals or nursing homes and so on, and this knowledge is of course worth its weight in gold for a relative, so that was a great help to me, and they are just so, it quickly built up on a friendly basis, somehow.”*

(A10, 50–55)

The personalization of care, the recognition of individual needs, and the building of consistent relational contact created a strong emotional anchor for both older adults and their family members. These relationships contributed significantly to feelings of being “seen,” “understood,” and “cared for”. The felt need for emotional security extended beyond the direct recipients of care to include informal caregivers, who found reassurance in the regular presence and proactive communication of the community nurses.

**Need for Flexible and Accessible Support**

Participants consistently emphasized the importance of flexible and low-threshold access to Community Nursing support. Unlike rigid institutional services, community nurses were perceived as available and approachable, enhancing clients’ sense of autonomy and control over their health and care.

Participants stressed the importance of being able to reach out without bureaucratic barriers:

*“That happens again and again, with a companion, I have community nurses in the game again, who are always there as experts, who I can contact at any time, long past the time that something like that is there […]”*

(A5, 807–810)

This accessibility was particularly important in building a sense of security:

*“If I have a question, I can call [the community nurse] at any time, at any time.”*

(A7, 144–145)

Participants contrasted this flexibility with previous experiences of restricted office hours or long waiting times when accessing municipal services or healthcare providers. The immediacy of contact, combined with the professional competence of the nurses, addressed a strong felt need for reliable, low-threshold health support.

**Need for Help Navigating Complex Health Information**

Another crucial felt need was assistance in understanding and managing complex health information. Older adults and caregivers often described being overwhelmed after consultations with doctors or hospital staff. Community nurses served as vital translators and interpreters of medical jargon and care plans.

Informal caregivers described the confusion and stress following healthcare encounters:

*“[…] because I also notice, so when someone has to go to the doctor, you’re excited, depending, because you’re so nervous that you can’t hear anything anymore, understand everything, and it’s also a wealth of information, what do I have to do now, mom very often said afterwards ‘you, what do I have to do now, what does the doctor want from me [spoken with question intonation]’“*

(A2, 392–396)

Community nurses were perceived not just as sources of information but as interpreters who could contextualize, simplify, and personalize health information according to the emotional and cognitive needs of older adults and their caregivers. This interpretive role enhanced participants’ feelings of competence and empowerment.

**Need for Support During Critical Life Transitions**

Participants articulated a strong felt need for support during critical life transitions, particularly hospital discharges. Transitions between healthcare settings often represent vulnerable periods characterized by uncertainty, physical weakness, and administrative complexity.

Community nurses played a key role in smoothing these transitions, offering emotional reassurance as well as practical support:

*“[…] and help directly at the hospital discharge and meet the patients, these are always stressful situations for the population groups, the first days, the first day after discharge, and I think to myself that there has definitely been, yes, a positive development.”*

(A6, 285–289)

Participants indicated that the early post-discharge phase was often when older adults were at their most vulnerable, both physically and psychologically. The presence of a familiar and trusted professional during this period was described as critical to maintaining health stability and preventing rehospitalization.

**Expressed Needs**

Expressed needs became visible through concrete actions such as service utilization, help-seeking behavior, and active engagement with Community Nursing services. The findings suggest that previously unmet needs were translated into concrete support-seeking practices once low-threshold and accessible structures became available.

**Active Help-Seeking and Communication with Community Nurses**

Participants consistently described community nurses as first-line contact points for a wide variety of questions, concerns, and situations. The ability to call or reach out to the nurses freely and without bureaucratic obstacles was highly valued, and participants made active use of this possibility.

One informal caregiver described the supportive nature of this communication:

*“Yes, the added value and benefit is clearly in the early or low-threshold possibility of seeing a qualified nurse, simply with a phone call or making an appointment, then she comes home to look at the living situation and can offer or make adaptations.”*

(A6, 76–79)

The low-threshold accessibility of community nurses enabled participants to seek support early, often before situations escalated into crises, thereby reinforcing the preventive function of Community Nursing in everyday care situations.

*“So I think the best case scenario is prevention, I would have said that I can make sure that people are looked after as well as possible at home, because then, if it doesn’t escalate at all”*

(A5, 385–388)

**Participation in Preventive Health Programs**

Several participants reported active participation in preventive programs initiated or supported by Community Nurses. These programs often involved physical activities, cognitive exercises, and social engagement—all essential to maintaining functional independence in older adults.

One network partner described the successful launch of such a program:

*“Doing a prevention programme with the elderly, with memory exercises, movement exercises, just all-round feel-good units, and we have launched a [health-promoting service] together with the Community Nurses.”*

(A7, 191–194)

The expressed need for such activities—and the high uptake by participants—underlines that preventive services are not merely “nice-to-have” additions but essential elements of aging well in the community. Participants’ engagement in these programs also highlighted the desire for community belonging and self-efficacy, as they actively took steps to maintain their own health and social integration.

**Seeking Support During Hospital Discharge and Post-Discharge Periods**

Participants actively sought the assistance of Community Nurses during hospital discharges and the immediate post-discharge phase:

*“[…] that you can involve an outside organization [Community Nurses] in certain communities, that will make the discharge for us, so we are less worried about the discharge, whether it works now or not at home, because I know the Community Nurse will come again.”*

(A6, 273–277)

One network partner reflected on the positive changes observed regarding re-admission cases due to the intervention of Community Nurses:

*“Yes, so those who are already being looked after by the Community Nurse, there is definitely a difference, I think that, firstly, we have fewer, so from the feeling, fewer referrals from these areas, because of course the Community Nurse is already active in the municipalities […]”*

(A6, 297–300)

Participants expressed gratitude for the presence and proactive support offered during these transitions, including health monitoring, emotional support, and practical guidance. This finding highlights that hospital-to-home transitions represent a critical juncture where expressed needs are high, and Community Nurses successfully fill a gap that traditional healthcare services often leave unaddressed.

**Assistance with Administrative Processes**

In addition to emotional and medical support, participants expressed a need for help with navigating administrative and bureaucratic tasks—particularly in relation to applications for care allowances or accessing services.

One participant described the substantial relief provided by Community Nurses:

*“[…] because the bureaucracy when someone wants to go into a care home or the bureaucracy when applying for care allowance or a pension is becoming increasingly difficult […]”*

(A7, 409–411)

This practical assistance was highly appreciated, as many participants found administrative processes confusing and burdensome, especially in emotionally charged contexts such as declining health. Community nurses’ support in this area reflects not only the practical meeting of expressed needs but also broader efforts to promote equity and reduce access barriers for older adults and their families.

**Comparative Needs**

Comparative needs became visible through contrasts between municipalities with and without Community Nursing services, revealing how access to support remained shaped by geography, mobility, and local infrastructure. Participants frequently compared current care situations with experiences prior to implementation, thereby highlighting perceived inequities in service availability and accessibility.

**Limited Access to Services Before Community Nursing**

Before the establishment of Community Nursing services, participants described a patchwork of informal support systems and sporadic municipal services. Formal healthcare or social support structures were either absent, minimal, or difficult to access, particularly for older adults with mobility restrictions.

One network partner illustrated the situation:

*“Yes, that’s perfect, that’s great, before the community nurses arrived in our region, we were not so well positioned in the district when it comes to support offers for senior citizens.”*

(A5, 167–169)

Participants described the need to rely on personal contacts, word-of-mouth networks, or informal organizations such as local parishes:

*“[…] it can be a parish, because someone there is socially committed, where you turn to, where you get the tip, you, go to that person and get another hint […]’“*

(A9, 326–328)

These informal systems were highly variable in quality, dependent on individual goodwill, and inaccessible to those without the right social connections.

**Geographic Inequities and Mobility Barriers**

Another dimension of comparative needs related to geographic disparities. Participants living in rural areas described significant challenges in accessing basic social and healthcare services, largely due to the physical distance to municipal centers and the lack of transport options.

One informal caregiver emphasized the exclusion created by distance:

*“They are really 30 kilometres away from the nearest social counselling centre, or 40 or 50, and he has no way of getting counselling there because he can’t get there at all.”*

(A5, 222–225)

Moreover, even when counselling services existed, their design often implicitly excluded those with physical or cognitive limitations:

*“The social counselling centre is responsible for this, but they don’t offer home visits, which means it’s only ever been possible for those who are mobile enough to have the cognition to do so.”*

(A5, 399–402)

The absence of home-based, proactive services for immobile or frail older adults constituted a systematic structural disadvantage—one that Community Nursing sought to address by bringing services directly into people’s homes.

**Improvements Perceived Through Community Nursing**

In areas where Community Nursing was implemented, participants reported improvements in empowerment for prevention. The availability of local, proactive care is seen as transformational, probably reducing the need for crisis interventions in the future.

A political decision maker noted the difference:

*“ […] and, of course, what we are now noticing more and more, which was not so pronounced at the beginning, that they also have this preventive approach, because classically, I say, there’s an accident, fall, illness, whatever, hospitalization, if necessary, and then the people come home and have to be looked after somehow, and that this prophylaxis, what could that look like, is set up a bit in advance so that not everyone is simply surprised, that’s what we are now increasingly aware of.”*

(A8, 88–96)

Participants directly attributed improvements in preventive care, informal caregiver support, and early intervention to the presence of community nurses, highlighting that previously unmet needs were now actively addressed. These improvements demonstrate that the comparative needs between municipalities can be reduced by implementing comprehensive, community-based services.

**Fears About Service Discontinuation and Call for Expansion**

Despite these successes, participants voiced strong concerns about the fragility of the new services, particularly regarding future funding and political support. Many participants feared that without permanent financial structures, the improvements achieved through the Community Nursing pilot project could be lost, thus recreating the inequities they had previously faced.

One network partner expressed this anxiety clearly:

*“I would find it a catastrophe if the project were to be scrapped as unsuccessful, I would find that very bad, that would be an indictment of Austria […]”*

(A5, 773–776)

One informal caregiver emphasized the personal and collective sense of security the service had provided:

*“For the future, for me it would be an insane slump if it were to disappear now, because it’s simply the support, it gives me stability now, it’s a kind of security for me, I always have the option of calling, and I think for the general public too, when they realize what’s on offer, they’ll be overrun anyway.”*

(A1, 363–376)

The call for expansion beyond the pilot regions was loud and clear:

*“[…] that we find a solution throughout Austria, yes, Community Nursing is now a federal issue anyway, yes, I do hope that they manage to do that [laughs], that it then becomes established throughout Austria.”*

(A5, 768–770)

*“[…] and outside of the municipalities, if someone needs it, it’s not possible to offer it, and that’s exactly what I see as a problem, so I think that if Community Nurses were used or could be used across the board, it would have a benefit for the general population, an added value.”*

(A6, 65–69)

These calls reflect an awareness among stakeholders that scaling Community Nursing services nationally would be crucial to reducing comparative inequities and ensuring equitable health outcomes for all older adults in Austria, regardless of place of residence.

Across all four need dimensions, a consistent pattern emerged: Community Nursing was not primarily perceived as a narrowly clinical service, but as a relational and coordinating infrastructure that compensated for fragmentation within existing health and social care systems. Stakeholders repeatedly emphasized continuity, accessibility, navigation support, and proactive engagement as central features of the service. While different stakeholder groups highlighted distinct dimensions of need, all groups described Community Nursing as filling gaps that previously remained unaddressed within traditional care structures. The findings further suggest that needs were shaped not only by individual health conditions but also by geographic context, service accessibility, social support structures, and the ability to navigate complex systems of care. Across stakeholder groups, normative needs were articulated primarily by network partners and local political decision-makers, reflecting their system- and structure-level vantage point on service organization and financing. In contrast, Felt and Expressed needs were more frequently voiced by informal caregivers, whose accounts centered on emotional security, trust, and the immediate, lived experience of giving or receiving care. Network partners and local political decision-makers, meanwhile, more often framed comparative needs in terms of service equity and sustainability across municipalities, while informal caregivers emphasized the personal consequences of potential service discontinuation. These patterns suggest that the four need dimensions were not experienced uniformly across groups, but were shaped by each group’s distinct relationship to Community Nursing—as direct recipients, professional collaborators, or system-level stakeholders. Beyond this overall pattern, three more specific relationships across subcategories were identified, as illustrated in Figure 1. First, the comparative need reflecting limited access to services before Community Nursing was directly addressed by Normative and Expressed needs concerning case tracking, administrative assistance, and navigation of complex health information, suggesting that Community Nursing was perceived as compensating for a previously fragmented system through concrete, practical support. Second, the Normative need for accessible preventive services, together with the Felt need for flexible and low-threshold support, translated into the Expressed need to actively participate in preventive health programs, which stakeholders in turn perceived as a tangible comparative improvement over the situation before Community Nursing. Third, the Felt need for trusting relationships underpinned participants’ expressed willingness to actively seek help and contact community nurses, while the same relational trust appeared to underlie the comparative fear that discontinuing the service would mean losing this source of security. These three patterns suggest that, rather than operating as four independent dimensions, normative, felt, expressed, and comparative needs were experienced as interconnected, with relational trust and practical navigation support functioning as recurring threads across categories.

## 4. Discussion

This qualitative study examined stakeholder-perceived needs during the early implementation of Community Nursing, using Bradshaw’s Taxonomy of Needs as an analytical lens. By integrating perspectives of informal caregivers, network partners, and local political decision-makers, the findings illuminate how different types of needs emerge, intersect, and evolve when a community-based nursing model is introduced into a traditionally hospital- and physician-centered healthcare system. Rather than focusing on outcomes or effectiveness, this study contributes an implementation-oriented perspective that highlights system fit, relational dynamics, and sustainability concerns from the viewpoint of those directly involved.

Across all four dimensions of need, stakeholders consistently described a pre-existing care landscape characterized by fragmented responsibilities, limited preventive services, and substantial geographic inequities. The introduction of Community Nursing was perceived as addressing several of these gaps simultaneously, particularly by creating continuity, low-threshold access, and relational stability. At the same time, participants expressed strong concerns about the long-term viability of the service, underscoring that early implementation success does not automatically translate into systemic integration.

**Normative Needs: Reframing Prevention, Continuity, and Responsibility**

Normative needs articulated by stakeholders centered on expectations regarding preventive care, continuous case tracking, advocacy, and organizational responsibility. These expectations reflect a broader recognition that curative, episodic healthcare alone is insufficient to meet the needs of an aging population living with increasing multimorbidity. Stakeholders did not merely describe Community Nursing as an additional service, but as a corrective structure addressing long-standing systemic blind spots.

In particular, the emphasis on proactive, ongoing engagement highlights a shift away from reactive service models toward anticipatory care. Continuity of contact was perceived as essential for early detection of deterioration, coordination across sectors, and sustained support for both older adults and their caregivers. This interpretation aligns with international findings indicating that relational continuity is a key mechanism through which preventive and community-based care can reduce avoidable crises and improve care coordination [27].

Importantly, normative needs also extended beyond professional roles to questions of governance and financing. Stakeholders repeatedly emphasized that municipalities alone could not sustainably shoulder responsibility for Community Nursing. These concerns point to a structural mismatch between local-level implementation and higher-level funding and regulatory frameworks, a challenge frequently observed in early-stage health system innovations [28]. The findings suggest that without clear regional or national anchoring, normative expectations risk remaining aspirational rather than actionable.

**Felt Needs: Relational Security as a Core Element of Care**

Felt needs in this study were predominantly relational and emotional in nature. Informal caregivers and older adults emphasized trust, emotional security, and the reassurance derived from consistent, personal relationships with Community Nurses. These relationships were not described as incidental but as central to participants’ sense of safety, confidence, and coping.

The findings underscore that relational continuity functions as more than a supportive add-on; it constitutes a core component of perceived care quality. Regular home visits, familiarity with individual life contexts, and sustained communication enabled participants to feel understood and accompanied over time. Such relational care has been shown to influence engagement with services, adherence to recommendations, and emotional well-being, particularly among older adults navigating complex health situations [29,30].

Notably, felt needs extended equally to informal caregivers, who described significant emotional relief in knowing that a trusted professional was regularly monitoring the situation. This highlights the dual focus of Community Nursing on both clients and their caregiving networks and suggests that emotional security operates at the level of the care constellation rather than the individual alone.

**Expressed Needs: From Availability to Active Use**

Expressed needs became visible through stakeholders’ active engagement with Community Nursing services. Participants did not merely value the availability of support but made concrete use of it—contacting Community Nurses for advice, participating in preventive programs, seeking assistance during hospital discharges, and requesting help with administrative processes.

The high level of utilization observed in this study suggests that unmet needs existed prior to the pilot project and that Community Nursing effectively lowered access barriers. Particularly during transitional phases, such as hospital discharge, Community Nurses were perceived as stabilizing actors who bridged gaps between institutional care and home-based living. This interpretation is consistent with evidence that transitional support and case coordination are critical points of intervention for preventing adverse outcomes in older populations [31,32].

Assistance with administrative and bureaucratic procedures further illustrates how expressed needs extend beyond clinical concerns. Navigating care allowances, services, and eligibility criteria was experienced as burdensome and stressful, and Community Nurses were valued as intermediaries capable of translating complex systems into manageable steps [33,34]. These findings reinforce the notion that effective community-based care must address social and administrative dimensions alongside health-related needs.

**Comparative Needs: Inequities, Place, and Access**

Comparative needs were most evident when participants contrasted care situations before and after the introduction of Community Nursing, as well as between municipalities with and without access to the service. These comparisons revealed pronounced geographic inequities, particularly affecting rural areas where distance, mobility limitations, and sparse service availability restricted access to care.

Participants’ accounts highlight how reliance on informal networks and ad hoc solutions created uneven and often inequitable support structures. The introduction of Community Nursing was perceived as mitigating some of these disparities by bringing services directly into people’s homes and reducing dependence on individual mobility or social capital. Similar patterns have been described in international research on rural health inequities, where place-based service models are essential for addressing structural disadvantage [35,36].

At the same time, the strong calls for expansion and fears of service discontinuation underscore that comparative needs are not static. Stakeholders were acutely aware that gains achieved in pilot regions could be lost if Community Nursing were not scaled and sustainably financed. This tension highlights the risk of reinforcing inequities through uneven implementation rather than alleviating them.

**Policy and Practice Implications**

Taken together, the findings suggest that Community Nursing addresses multiple dimensions of need simultaneously, positioning it as a potentially transformative component of community-based care. However, the study also indicates that successful early implementation must be accompanied by clear policy commitments, stable funding mechanisms, and explicit role definitions to prevent fragmentation and insecurity.

The advocacy role attributed to Community Nurses points to the need for policy frameworks that formally recognize nursing leadership in prevention, coordination, and health system navigation. Aligning professional mandates with governance structures would support the transition from pilot initiatives to integrated services and reflect international calls to strengthen nurses’ roles in shaping health systems [8].

**Strengths, Limitations, and Future Research**

This study provides in-depth qualitative insights into an early-stage Community Nursing initiative, capturing diverse stakeholder perspectives during a critical implementation phase. The use of Bradshaw’s Taxonomy of Needs enabled a structured yet flexible analysis that links individual experiences to systemic considerations. At the same time, the a priori application of Bradshaw’s four-category structure may have shaped both the organization of the data and the interpretive lens through which stakeholder accounts were understood, potentially foregrounding certain dimensions of need over others that a purely inductive approach might have surfaced differently. The study’s contribution lies not only in applying this established taxonomy, but in its implementation-phase, multi-stakeholder perspective, which has received comparatively limited attention in prior community nursing research.

Nevertheless, the study has limitations. The sample size was small (*n* = 11) and regionally bounded, which limits transferability. However, the inclusion of heterogeneous stakeholder groups and the focus on information-rich cases enhance the analytic depth and relevance of the findings. Future research should include longitudinal designs to examine how perceived needs evolve over time and comparative studies across regions and countries to explore contextual influences on implementation success. Several additional limitations warrant mention. First, participants were recruited with the assistance of community nurses already known to the first author through prior interviews within the same project; while these community nurses had no influence on interview content, their role in facilitating access to participants is acknowledged as a potential source of selection bias. Second, individuals who volunteer to be interviewed about a pilot project they are personally engaged in may be more favorably disposed toward it, which may have limited the visibility of more critical perspectives in the dataset and contributed to the predominantly positive tenor of the findings. Third, member checking (participant validation of the findings) was not conducted, which limits the extent to which participants could confirm or contest the credibility of the final interpretation. Future studies could address these limitations through independent recruitment channels, purposive inclusion of participants with more critical views, and structured member-checking procedures.

Finally, it is important to note that following data collection (October 2023–March 2024), national and EU-level financing for Community Nursing in Austria ended in December 2024, with responsibility devolved to the counties [23]. The findings therefore reflect stakeholder experiences prior to these decisions and underscore a clear demand for sustained and secure funding structures.

## 5. Conclusions

This qualitative study highlights how Community Nursing addresses multiple stakeholder-perceived needs during early implementation, including preventive care, relational continuity, accessibility, and navigation support. Using Bradshaw’s Taxonomy of Needs, the findings show that Community Nursing fills critical gaps in fragmented, hospital-centric care structures, particularly for older adults and informal caregivers in rural settings.

Stakeholders valued the relational, low-threshold nature of Community Nursing and its role in promoting trust, continuity, and proactive support. At the same time, concerns regarding long-term financing and governance underline the vulnerability of pilot-based initiatives without systemic integration.

Overall, the findings suggest that Community Nursing has the potential to contribute meaningfully to preventive, community-based care if supported by stable organizational and funding frameworks. Given the small, context-specific sample, these findings should be interpreted as offering context-bound insights rather than broadly generalizable conclusions; nonetheless, they may inform health systems seeking to strengthen community nursing models in response to demographic ageing and increasing care complexity, particularly in comparable early-implementation settings.

## Figures and Tables

**Table 1:** Anonymized participant characteristics (N = 11).

ID	Gender	Age	Stakeholder Group	Interview Mode	Duration (min)
A1	Female	45	Informal caregiver	Telephone	34:07
A2	Female	64	Informal caregiver	Zoom	57:12
A3	Female	48	Network partner	Telephone	28:57
A4	Female	76	Informal caregiver	Telephone	40:29
A5	Female	45	Network partner	Zoom	67:27
A6	Female	32	Network partner	Telephone	36:48
A7	Female	63	Informal caregiver	Telephone	35:44
A8	Male	46	Local political decision-maker	Telephone	32:36
A9	Male	66	Local political decision-maker	Zoom	35:07
A10	Female	58	Informal caregiver	Telephone	55:26
A11	Female	57	Local political decision-maker	Zoom	35:29

## Data Availability

The data presented in this study are not publicly available due to privacy and ethical restrictions. De-identified excerpts of the data may be available from the corresponding author upon reasonable request and with permission of the Ethics Committee.

## Supplementary Materials

The following supporting information can be downloaded at: https://www.mdpi.com/article/10.3390/nursrep16070228/s1, COREQ Checklist (Consolidated Criteria for Reporting Qualitative Research).

## Public Involvement Statement

No public involvement in any aspect of this research beyond participation as study subjects.

## Guidelines and Standards Statement

This manuscript was prepared in accordance with the COREQ (Consolidated Criteria for Reporting Qualitative Research) guidelines for qualitative interview studies.
