# Supplementary material for: Stakeholder-Perceived Needs in Early Community Nursing Implementation: A Qualitative Study
Source: Nurs Rep. 2026 Jun 30;16(7):228. doi: 10.3390/nursrep16070228 (PMC13414509; doi:10.3390/nursrep16070228)
Supplement: Supplementary file 1 [file nursrep-16-00228-s001.zip › nursrep-4345366-supplementary.pdf]

COREQ Checklist (Consolidated Criteria for Reporting Qualitative Research)

Domain 1: Research team and reflexivity

Item	COREQ Item	Addressed in Manuscript
1	Interviewer/facilitator	All interviews were conducted by the first author (C.F.), a nursing researcher (Methods, Section 2.4).
2	Credentials	C.F. is a nursing researcher; co-authors' professional backgrounds include nursing, public health, medicine and health services research (H.B., I.G., M.T.).
3	Occupation	C.F. (nursing researcher); H.B., I.G., M.T. (nursing/public health researchers/medical professionals); see Author Contributions.
4	Gender	Gender of participants are outlined in table 1. Gender of authors is C.F. female, M.T. female, H.B. male and I.G. male.
5	Experience and training	all authors have recieved formal training in qualitative research methods and have extensive experience in qualitative research. C.F. H.B. and M.T. are experienced nursing professionals and resaerchers, M.T, I.G. is a phyisican and a public health researcher.
6	Relationship established	The interviewer (C.F.) had no prior personal relationship with any of the participants (Methods, Section 2.4). Participants were recruited with the assistance of community nurses already known to C.F. through prior project-related interviews; this is acknowledged as a potential source of selection bias (Strengths and Limitations).
7	Participant knowledge of the interviewer	Participants were informed about the study aims, the researcher's role, confidentiality, and voluntary participation prior to the interview (Methods, Section 2.3).
8	Interviewer characteristics	Nursing researcher, PhD student, trained nurse with some years of practical experience. – Relevant aspects (nursing researcher, mentioned in script).

Domain 2: Study design

Theoretical framework

Item	COREQ Item	Addressed in Manuscript
9	Methodological orientation	Interpretive descriptive qualitative study using qualitative content analysis (Kuckartz & Rädiker), theoretically informed by Bradshaw's Taxonomy of Social Need (Methods, Section 2.1, 2.5).

Participant selection

Item	COREQ Item	Addressed in Manuscript
10	Sampling	Purposive sampling via existing project networks (community nurses facilitating contact); exploratory, information-rich case sampling rather than independent saturation per subgroup (Methods, Section 2.3).
11	Method of approach	Participants were contacted with the assistance of community nurses involved in the pilot project (Methods, Section 2.3).
12	Sample size	11 participants across three stakeholder groups: informal caregivers, network partners, and local political decision-makers (Table 1).
13	Non-participation	Not separately reported; no systematic refusals were documented.

Setting

Item	COREQ Item	Addressed in Manuscript
14	Setting of data collection	Interviews were conducted via telephone or online video call (Zoom), depending on participant preference (Methods, Section 2.4).
15	Presence of non-participants	Not applicable; no third parties were present during interviews.
16	Description of sample	Gender, age, stakeholder group, interview mode, and interview duration for all 11 participants are presented in Table 1 (Section 2.3).

Domain 3: Data collection

Item	COREQ Item	Addressed in Manuscript
17	Interview guide	Three stakeholder-specific semi-structured interview guides were developed collaboratively by several members of the research team prior to data collection (Methods, Section 2.4).
18	Repeat interviews	Not conducted; each participant was interviewed once.
19	Audio/visual recording	All interviews were digitally audio-recorded with consent and transcribed verbatim (Methods, Section 2.4).
20	Field notes	A research diary was maintained, with reflective notes recorded after each interview, documenting contextual observations and notable circumstances (Methods, Section 2.4).
21	Duration	Interview duration ranged from 28 minutes 57 seconds to 1 hour 7 minutes 27 seconds; durations for all participants are reported in Table 1.
22	Data saturation	The study was designed as an exploratory investigation without an aim to achieve independent saturation within each stakeholder subgroup. Data collection and coding proceeded iteratively; from the ninth interview onward no substantially new subcategories emerged, and two further interviews were conducted to confirm this pattern (Methods, Section 2.3).
23	Transcripts returned	Not conducted; this is acknowledged as a limitation (absence of member checking/participant validation) (Strengths and Limitations).

Domain 4: Analysis and findings

Item	COREQ Item	Addressed in Manuscript
24	Number of data coders	Coding was conducted by the first author (C.F.). Inductive subcategories and their alignment with Bradshaw's deductive main categories were discussed and reflected upon with a co-author (H.B.) as a form of peer debriefing (Methods, Section 2.5).
25	Description of coding tree	Deductive coding based on Bradshaw's Taxonomy of Social Need (normative, felt, expressed, comparative needs), combined with

Item	COREQ Item	Addressed in Manuscript
		inductive sub-category development from the empirical material (Methods, Section 2.5; Figure 1).
26	Derivation of themes	Themes (subcategories) were derived through an iterative deductive–inductive analytical process, with analytical memos documenting coding decisions and providing an audit trail (Methods, Section 2.5).
27	Software	MAXQDA, version 22.7.0 (Methods, Section 2.5).
28	Participant checking	Not conducted; acknowledged as a limitation. Peer debriefing with a co-author (H.B.) was used as an alternative trustworthiness strategy (Methods, Section 2.5; Strengths and Limitations).
29	Quotations presented	Illustrative participant quotations are provided throughout the Results section, attributed by anonymized participant ID (e.g., A1–A11).
30	Data and findings consistency	Quotations are presented alongside analytic interpretation for each subcategory; consistency between data and reported findings is maintained throughout the Results section.
31	Clarity of major themes	The four major need dimensions (normative, felt, expressed, comparative) are clearly presented and illustrated in Figure 1.
32	Clarity of minor themes	Subcategories within each need dimension are clearly presented; identified cross-cutting relationships among subcategories are additionally discussed in the Results and illustrated in Figure 1.

Note: This checklist reflects the methodology and reporting as presented in the revised manuscript (track-changes version) and the accompanying point-by-point responses to reviewers.
